# A clinical evaluation of setup errors for a prostate immobilization system

**DOI:** 10.1120/jacmp.v1i4.2635

**Published:** 2000-09-01

**Authors:** John E. McGary, Walter Grant

**Affiliations:** ^1^ Department of Radiology Baylor College of Medicine Houston Texas 77030

**Keywords:** immobilization, setup errors, prostate

## Abstract

A prostate treatment immobilization system was evaluated with respect to setup errors and efficiency for a specific treatment setup. Prostate patients were treated in the prone position with a rectal catheter using the NOMOS intensity modulated radiotherapy system. Immobilization and setup consisted of a Vac‐Lok™ bag (MED‐TEC, Orange City, IO) fitted within a registration carrier box where patients were aligned to the bag using skin marks along the lower leg. Daily setup errors were analyzed using lateral portal films, registration plates mounted to the carrier box, and the pubic symphasis as a bony reference. Two studies were conducted to evaluate setup technique. In the first study, patient setup required 3–5 minutes for patient positioning and the corresponding superior/inferior errors were found to have a standard deviation of 3.5 mm. In the second study, the technique standards were reduced to allow for faster setup times and, consequently, larger errors; setup times were 1–2 minutes and the mean and standard deviation errors were ~2 and 5 mm, respectively.

PACS number(s): 87.53.–j, 87.90.+y

## INTRODUCTION

The goal of conformal radiotherapy is to minimize the dose to normal tissue surrounding the clinical target volume (CTV). Any deviations between the planned and treatment position degrade the therapeutic ratio. The planning target volume (PTV) is defined to include the CTV and associated treatment uncertainties, which include but are not limited to imaging, patient setup, and organ motion. For conditions where planning margins are not sufficient, the tumor will be underdosed. In contrast, margins that are too large may lead to greater complications. While it is impossible to eliminate these errors, the goal is to measure the planning margins for a specific clinical environment and reduce the uncertainties where possible.

Prostate motion, defined as a positional change of the prostate at the time of treatment relative to the planning position, has been evaluated for different conditions and methods.^1^ ^–^ ^10^ Radio‐opaque markers, gold seeds, CT‐CT fusion, and CT chamfer matching are examples of methods used to determine the prostate position relative to fixed bony landmarks. Within these studies, the largest motion was observed to be in the anterior/posterior (A/P) direction and to a lesser extent in the superior/inferior (S/I) direction. The variation in A/P data between reports is considerable. Values of A/P shifts range from (–0.9 mm mean, 1.7 mm standard deviation) to (–5.4 mm mean, 6.2 mm standard deviation). The range in S/I shifts were (–0.2 mm mean, 3.2 mm standard deviation) to (–5.9 mm mean, 5.0 mm standard deviation). The lateral displacements were shown to be minimal—the mean and standard deviations were less than 1.5 mm. In addition to measuring organ movement, correlations were made for bladder and rectal filling, rectal contrast, and random bladder volumes. The A/P movement was strongly correlated with rectal filling and then to a lesser extent with bladder filling. In the case of patients treated in the prone position, the bladder effect was correlated with both A/P and S/I movement.

Along with prostate motion studies, patient setup accuracy was investigated to establish margins for planning target volumes. Setup errors were determined for different treatment conditions: supine, prone, immobilized, and unimmobilized. The studies typically showed that treatment accuracy is increased by immobilization techniques and setup correction strategies.^11^ ^–^ ^22^ Currently, the consensus is that patient setup accuracy is better with immobilization although some investigators report insignificant improvement.^12^ For unimmobilized patient setup, the standard deviation of setup errors for prostate patients was reported to range from 2.1–3.8, 2.5–3.0, 2.5–5.5 mm for the lateral, superior/inferior, anterior/posterior directions.^11^ ^–^ ^14^ ^,^ ^22^ For patients with immobilization casts, the mean displacement and the random component characterized by the standard deviation was less than ~2 mm, which is small compared with organ motion.^11^ ^,^ ^12^ Most of the analysis was based on daily portal films or electronic portal images, although some authors used retrospective analysis of weekly port films. A current summary of setup errors and organ motion results is found in Antolak *et al*.^23^

The purpose of this paper is to evaluate setup errors and a correction method for a prostate immobilization system where patients are treated in the prone position with a rectal catheter. A simple system to determine setup deviations required for treatment planning and the factors affecting patient positioning and efficiency is also described.

## METHODS AND MATERIALS

### Patient setup

**Figure 1:**
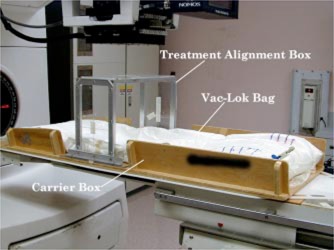
(Color) The prostate carrier box with Vac‐Lok bag and treatment alignment box.

Patients were set up in the prone position and immobilized using a VacLok™ bag (MED‐TEC, Orange City, IO) which was fitted to a plywood box designed for registration and support. The carrier box was designed to maintain the shape of the Vac‐Lok bag for patient repositioning and prevent breakdown over extended periods of time. Side walls along the box provided lateral support for the vacuum bag and contributed as hand rails for entering and exiting the box. Inside the box, a 1/4‐in.‐thick Lucite registration plate was mounted to the base. This Lucite‐base plate consisted of a **Z** fiducial system to define the sagittal center of the box and determine lateral and longitude offsets. In addition, the plate ridge suppressed bag movement along the superior/inferior direction. On both sides of the Lucite‐base plate, dowel pins were fixed to hold removable CT and portal film‐registration fiducial plates or a treatment alignment box. The treatment alignment box was an aluminum U‐shape frame that held three Lucite plates, two along the sides of the frame and one for the top (Fig. 1). The top plate allowed for sagittal alignment with lasers or light‐field cross hairs, whereas the side target plates were used for vertical, longitudinal, and rotational alignment.

**Figure 2:**
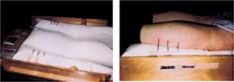
(Color) Marks on bag (left) and patient setup according to marks on legs and bag (right).

Before the patient arrived for the planning CT, the Vac‐Lok bag was preformed as follows: the bag was doubled over to reduce bag volume to snuggly fit the carrier box; the bag fill material was moved away from the center to the leg and chest area to provide better patient support; shallow troughs for the legs were formed to initiate the appropriate depth and entry path; and the bag material was formed against the half‐round, wooden dowels to prevent bag movement. For the planning CT, the patient entered the box with a preformed Vac‐Lok bag and was coached into position using lateral scout images to center the pubic symphasis in the superior/inferior direction with respect to the box fiducial system. After centering the patient longitudinally, the vacuum was released slightly to loosen the fill material for molding the bag to the patient contour. While the vacuum was being drawn, the bag was formed along the patient body. Forming the bag around the legs was critical since leg marks were used for daily treatment positions. If the leg troughs were too deep, skin attached to the bag and created a differential motion with respect to the bones. The bag was formed with enough depth to create pockets for the knees and allow for the marks on the bag to coincide with skin marks on the legs. To reduce skin contact with the bag, the bag was pulled laterally away from the calves during formation and the legs were rolled laterally to prevent skin adherence. After forming the bag to the patient, very small marks were made on the legs and bag for reference. The patient was removed from the bag and allowed to rest for about 5 minutes. After resting, the patient climbed back into the box and repositioned according to feel, and then was coached into position using the small marks on the bag and legs. This allowed the patient to become familiar with the bag and break down hard ridges that formed during the process. After the patient settled into position, a rectal catheter (a barium enema tip typically used for radiological procedures) was inserted and inflated to 100 cc. Approximately 5–10 minutes were allowed to pass to relieve the unsuspected and disturbing sensation of the catheter before performing the CT. The goal was to relax the patient as much as possible. After the CT was performed, the patient's legs were rolled laterally, and three continuous, vertical marks were drawn from the calves to the bag. Loose skin along the calves or incomplete bag contact was avoided for marking. Pictures of the leg marks for both legs were recorded for treatment setup. Figure 2 illustrates the marks on the bag with and without a patient.

**Figure 3:**
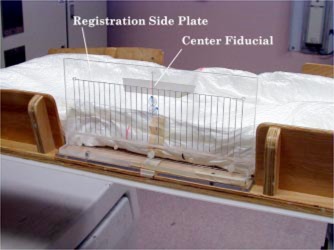
(Color) Registration side plate mounted on carrier box.

During CT, two registration side plates, each with one pair of horizontal and vertical wires, were attached to the box to identify the vertical and longitudinal zero planning position. The sagittal zero position was defined by the fiducial system on the Lucite‐base plate. After the axial images were acquired, one registration plate was replaced by a portal registration side plate that contained 20 vertical wires, with each wire physically separated by 1 cm, and one horizontal wire (Fig. 3). To avoid CT artifacts and maintain visibility during portal films, stainless steel wire of 0.032 in. diameter was embedded within 1/4 in. of Lucite to create the registration plates. For treatment verification, a lateral scout image was taken to register the pubic symphasis with daily portal images. If digital reconstructed radiographs (DRR's) were available, the portal registration plate was inserted for the treatment planning scan. From the scout or DRR, the vertical wire on the registration side plate with the least S/I distance from the pubic symphasis projection was recorded. For daily portal films, the patient was filmed with the recorded wire in the center of the field.

**Figure 4:**
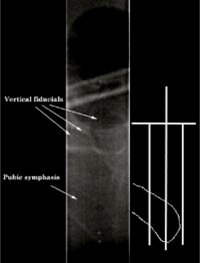
Portal image of pubic symphasis and registration plate with all MIMiC vanes open (center). The drawing illustrates the pubic symphasis and fiducial wires seen on the portal image (right).

Patients were treated with the NOMOS MIMiC (multileaf intensity modulating collimators‐NOMOS Corp., Sewickley, PA) radiation delivery system. Patients climbed into the prostate box, which was set on the treatment table, and were coached into position according to leg/bag marks and pictures from the original planning setup. The legs were rolled laterally to loosen skin contact and the final position was confirmed with setup pictures made during the original CT scan. The rectal catheter was then inserted and inflated. After registering the patient to the box, the box was aligned to the treatment‐room lasers using the treatment alignment box. After alignment, the leg marks were checked again for movement. For setup verification, a lateral portal image was taken with KODAK EC‐L high‐contrast film with all the MIMiC vanes open, which projected an image at isocenter of roughly $20\times 4\;{\text{cm}}^{2}$ that included the pubic symphasis and plate wires. Figure 4 is an example of a portal image used for verification where the actual image is in the center of the figure. To the right of the image is an illustration to help identify the pubic symphasis and fiducials that may not be as clear as in the original film. The bony landmarks are difficult to see on the first viewing but become easily recognized with some practice.

The registration plates require multiple embedded vertical wires due to the narrow MIMiC open‐field size used for portal films. Using a single vertical fiducial to identify the longitude center of the box would reduce the flexibility and time efficiency of the process. For patients planned with a separation distance greater than 2.5 cm between a center fiducial and pubic symphasis, setup errors greater than 1 cm would not be registered. To ensure that patients were always within 2.5 cm of the fiducial, multiple repositions would be required. In addition, taller patients could not be registered using a single vertical wire since they need to be more inferior in the box to avoid collision with the treatment machine gantry. For analysis, multiple wires spaced at 2 cm would be sufficient to register setup errors; however, the spacing would be too wide to estimate pre‐ or post‐treatment correction by visual inspection. From experiment, it was determined that 1 cm spacing between fiducials along the registration side plate allowed quick estimates of setup errors within 2 mm of measurement.

### Image registration

The pubic symphasis was registered to the box, using the registration side plate, to identify setup errors relative to the immobilization device. Box alignment errors were considered independent for defining the PTV. Daily lateral portal films of 30 patients were analyzed for this report. A majority of patients in this study received 35 treatment fractions while a few patients received between 10–20 fractions. Due to various reasons, films were acquired for about 90% of the daily treatments. The average number of films analyzed per patient was approximately 25. Patient setup errors in the superior/inferior direction were analyzed by comparing portal films, representing the actual treatment position, with the corresponding CT scout film or DRR, representing the planned treatment position. The pubic symphasis was outlined on each portal film and compared with the planned treatment position using the fiducial system on the portal plates as a method for registering the patient position with the box. Using a prostate phantom and several patient studies, the accuracy of the method was estimated to be ~2 mm.

## RESULTS

This study was restricted to superior/inferior setup errors since we found these to be larger than other sources of setup errors. A limited study involving four patients was used to estimate 3D setup errors using CT images and the Portal Imaging Processing System (PIPS, Masthead Imaging Corporation, Nanaimo, BC) registration software package. Patients were scanned twice per week for seven weeks. The prostate gland and surrounding bony anatomy were outlined on all images for comparison with the planning CT images. Registration to the planning position was made using the box registration fiducials and pubic symphasis. From these studies, the maximum lateral and anterior/posterior setup error was found to be within the range of ~4–6 mm. Daily lateral portal films confirmed the anterior/posterior range except for patients that used pillows or foam inserts to avoid pain from prior chest surgery. Under those conditions the anterior/posterior displacement error was observed to be a maximum of ~7–10 mm.

In addition, organ motion was estimated from the CT studies of the four patients. The prostate was outlined for each CT scan and compared with bony landmarks. These studies were an attempt to identify the largest displacement to be expected as a conservative approach for defining the organ margin. The limited study showed maximum prostate displacements of approximately 5–6 mm, which is consistent with the literature for the superior/inferior and lateral shifts.^23^ The anterior/posterior displacement measurements were found to be less by approximately 3–4 mm than many values reported in the literature and that is attributed to the rectal balloon. More recent studies involving patients with gold seed implants have shown less organ motion than the previous study. Three patients with gold seed implants were imaged with CT on 10 different days during the course of IMRT treatment. In these cases, the daily CT scans were fused to the CT planning scan. The maximum A/P and R/L organ motion was found to be ~2 mm while the maximum S/I displacement was observed to be ~3 mm. In addition, breathing studies show little movement, ~1 mm displacement, over a 5–minute period. These studies indicate that we may be overestimating the organ margin but more detailed studies are required to accurately define the PTV.

This study was divided into two groups to demonstrate technique differences. The first study included 16 patients that were closely monitored during setup whereas the second study involved 14 patients where the setup was loosely controlled. The first study was an attempt to identify proper technique, understand factors relating to setup errors, and decide upon error correction methods. For planning CT, one physicist formed the bag, marked the patient, and monitored the CT process. The same physicist was in the treatment room three out of five days to perform patient setup, record patient movements during treatment, take daily pictures for setup correlation, and compare portal films to planning position films. Two other physicists performed setup without pictures for the remaining two days of the week. Typically, setup time per patient was approximately 5 minutes, excluding the time required for portal films. Most of the effort was spent in examining leg marks and verifying with setup pictures. Patients were taken to CT for remarking when they were unable to setup within 7 mm for three consecutive fractions.

In the second study, patients were set up without using a specific or consistent technique; pictures of leg marks were not used to register patients to the bag, bag formation and marking were less precise, and close alignment of leg and bag marks was ignored. Methods for setup and bag formation resembled methods typically used in conventional radiotherapy treatment. The second study emphasized setup time. The patient load was increased to 25–30 patients treated per day as compared with Study I, where six patients were treated in the afternoon. Patient setup time decreased from approximately 5 minutes to 1 minute. Corrections to patient marks were made after the first 5 fractions if the setup error was 10 mm or greater for 3 fractions.

A summary of superior/inferior setup deviations between the two studies is shown in Table I. Approximately 400 portal films from each study were used for the analysis of setup errors. The collection of setup errors in each study represented a normal distribution with 95% of the values contained within two standard deviations of the distribution. The major difference between the two setup techniques is characterized by the standard deviation (i.e., the random setup error) of the total setup error distribution. The mean of the total setup error distribution (the systematic setup error) in these studies was on the order of the measurement uncertainty and considered to be insignificant with respect to the random component. In terms of the planning target volume, the margin may be determined from the standard deviation. The standard deviation of Study II is significantly larger, by ~2 mm, than found in Study I.

**Table I:** Summary of S/I setup errors for the total setup error distribution.

	Mean (mm)	Standard Deviation (mm)
Study I	0.43	3.54
Study II	‐1.77	5.23

**Figure 5:**
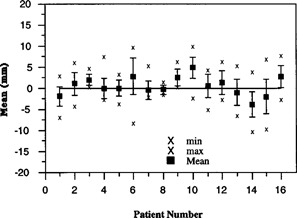
Setup error for Study I.

**Figure 6:**
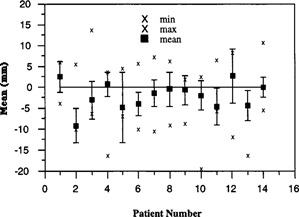
Setup error for Study II.

A more detailed comparison between studies is shown in Figs. 5 and 6. The figures show the mean setup error for each patient. To represent the random variations for each patient, the standard deviation is plotted as error bars about the mean values. The maximum and minimum values are included for perspective. In Study II, roughly 60% of the patients have at least one setup error greater than 1 cm and 20% of the patients have setup errors greater than 1.5 cm. In comparison, there is one displacement greater than 1 cm in Study I. Overall, the results of Study II illustrate larger random and maximum setup errors as compared with Study I. Even though one patient in each study was corrected one time, the results are not significantly different than if these patients were not included within the analysis.

## DISCUSSION

Different factors were found to affect patient setup such as the quality, location, and number of patient marks; Vac‐Lok fill density; consistent chest, feet, and head positions; and the use of setup pictures. The setup described in the paper was the result of testing different techniques to decrease setup errors at the expense of extended treatment time. For better setup, an additional 3–5 minutes per patient was required which extended the workday by one to two hours. We found that setup errors decreased as more attention was placed on bag formation and leg marking. In general, marks on the calves improved setup in comparison with marks on the thighs and pelvic area. Setup pictures provided an accurate reference to the planning position and enabled precise leg positioning in conjunction with rolling the patient's legs. The first study is a sample of acceptable setup errors for prostate treatment. Better setup will likely require more time and may not significantly reduce errors. The second study represents setup errors associated with the minimum setup time for prostate treatment—it will be difficult to position patients quicker than those in the second study. The second study demonstrates the compromise between setup time and error where setup errors are comparable to unimmobilized patient setup.

With respect to a study by Hanley *et al*., where prostate patients were immobilized and treated in the prone position, the setup errors found in Study I are ~2 mm larger than the reported values.^12^ Hanley *et al*., used alpha cradle casts, tattoo marks, and lasers for patient alignment.

They reported setup errors as 0.4 mm and 1.4 mm for the mean and standard deviation in the superior/inferior direction. The major differences between the two setup methods are the type of immobilization material and the use of rectal catheters. It is not known yet if the rectal catheter increases setup error. For a few of our pelvic prostate intensity modulated radiotherapy (IMRT) patients treated without rectal catheters, the setup errors were small, with standard deviations less than ~2 mm; however, the sample of patients was too small to resolve this as a major contributor to setup errors.

For conventional fields, skin marks coinciding with field edges are used for patient alignment. With the system used for our prostate IMRT patients, the patients are registered to the box using skin marks with the box aligned to the lasers. To date, we have not attempted to use patient marks for laser alignment as in conventional setup. In addition to the current use of leg marks, a mark on the small of the back for laser alignment may prove to be a better method for setup.

The portal registration plates were used as a simple method for measuring daily setup errors for creating planning target volume margins. With this system and procedure described earlier, we determined the setup errors in the superior/inferior direction. The results from a subsequent study of 50 patients were similar to Study II. Random setup errors in the S/I direction for the total population were found to have a standard deviation of 5.2 mm. A margin of 10.4 mm, 2 standard deviations, was used for the S/I setup error.

For setup verification, daily portal films were instituted since random setup errors dominated the systematic errors. Due to various problems, only ~300 patients received portal films for more than 80% of the treatment fractions with the goal of one portal film per fraction. Typically, there were no time trends associated with the daily portal films; patients that were setup within 5 mm for 60–70% of the time would occasionally setup ~1 cm away from the planning position. With daily patient loads between 25–35, the time required to film, review, and maintain, setup errors were substantial. As a consequence, the number of portal films per patient was reduced due to the lack of resources. Patients then received daily portal films for the first five treatment fractions and were re‐marked if 3/5 of the errors were ~1 cm. After the first five fractions, patients were filmed twice a week to control large displacements. With plate wires separated by 1 cm, the setup error was quickly estimated by comparing the location of the pubic symphasis between the lateral portal‐and scout film or DRR.

The prostate planning target volume was determined from a combination of our measurements and reported values. Patient setup errors in the A/P and the R/L directions were taken as the average of our CT registration measurements and values reported by Hanley *et al*., where patients were immobilized in a similar manner. Setup errors in the S/I direction were determined from our daily portal films using the method described in the paper. We chose two standard deviations for the setup margin since this included about 95% of the measured setup displacements. For organ motion, the average of the values reported in the literature were used for the S/I and R/L directions.^11^ The A/P prostate motion estimated from our CT studies showed less displacement than many values reported in the literature which is due to the rectal balloon. For CORVUS treatment planning system (NOMOS Corp., Sewickley, PA), the planning target volume is defined by localization and immobilization uncertainties. Immobilization uncertainty, which relates to the fixation device, is established by three user‐supplied values for the A/P, R/L, and S/I directions. The planner treats the patient as a rigid body and applies translational motion for all organs. Localization uncertainty accounts for independent organ motion with six possible directions (A, P, S, I, R, and L). Localization values are allowed for all targets and structures. The total uncertainty for the PTV is calculated by CORVUS as ${[{\left(\text{localization error}\right)}^{2}+{\left(\text{immobilization error}\right)}^{2}].}^{0.5}$ For our prostate PTV, we consider uncertainties in patient setup, box alignment, and image resolution as one value for the CORVUS immobilization uncertainty by summing in quadrature. Setup errors are considered independent of box alignment errors that are measured as ~1 mm standard deviation for all directions. Image uncertainties are estimated from the planning CT axial resolution for the A/P and R/L directions, and one half the slice thickness for the S/I uncertainty. Localization uncertainty is restricted to prostate motion only. A summary of values for the CORVUS prostate planning target volume are shown in Table II.

**Table II:** Summary of values used for CORVUS uncertainties (2×standard deviation)

	S/I (mm)	A/P (mm)	R/L (mm)
Patient setup	10.4	4	4
Box alignment	2	2	2
Image resolution	2.5	0.7	0.7
**Immobilization**	**10.9**	**4.5**	**4.5**
**Localization**	**6**	**5**	**2**
**Total Uncertainty**	**12.4**	**6.7**	**4.9**

## CONCLUSION

A prostate treatment immobilization system was evaluated with respect to setup errors and efficiency for a specific treatment setup where patients are treated in the prone position with a rectal catheter. Setup errors were correlated with technique and time. The best setup required 3–5 minutes for patient positioning and the corresponding superior/inferior errors were found to have a standard deviation of 3.5 mm. Daily portal films showed that patients would occasionally setup ~1 cm away from the treatment planning position even though most of the setup errors were closer to 5 mm. Reducing technique standards allowed for faster setup with larger errors; setup times were 1–2 minutes and the mean and standard deviation error were ~2 mm and 5 mm, respectively.

The overall system was fairly simple to fabricate and easy to implement. The portal registration plates were used to accurately register the patient planning position to the daily treatment position. Data analyzed from the portal films were used to estimate superior/inferior, and to a lesser extent anterior/posterior, setup errors necessary for establishing planning target volume margins. In addition, the plate system was used to control setup errors for routine treatment.
